# Effects of *MIG1*, *TUP1* and *SSN6* deletion on maltose metabolism and leavening ability of baker’s yeast in lean dough

**DOI:** 10.1186/s12934-014-0093-4

**Published:** 2014-07-04

**Authors:** Xue Lin, Cui-Ying Zhang, Xiao-Wen Bai, Hai-Yan Song, Dong-Guang Xiao

**Affiliations:** 1 Key Laboratory of Industrial Fermentation Microbiology, Ministry of Education, Tianjin Industrial Microbiology Key Laboratory, Tianjin University of Science and Technology, Tianjin 300457, PR China; 2 College of Biotechnology, Tianjin University of Science and Technology, Tianjin 300457, PR China

**Keywords:** Baker’s yeast, Glucose repression, MIG1, TUP1, SSN6, Maltose metabolism

## Abstract

**Background:**

Glucose repression is a global regulatory system in baker’s yeast. Maltose metabolism in baker’s yeast strains is negatively influenced by glucose, thereby affecting metabolite productivity (leavening ability in lean dough). Even if the general repression system constituted by *MIG1*, *TUP1* and *SSN6* factors has already been reported, the functions of these three genes in maltose metabolism remain unclear. In this work, we explored the effects of *MIG1* and/or *TUP1* and/or *SSN6* deletion on the alleviation of glucose-repression to promote maltose metabolism and leavening ability of baker’s yeast.

**Results:**

Results strongly suggest that the deletion of *MIG1* and/or *TUP1* and/or *SSN6* can exert various effects on glucose repression for maltose metabolism. The deletion of *TUP1* was negative for glucose derepression to facilitate the maltose metabolism. By contrast, the deletion of *MIG1* and/or *SSN6*, rather than other double-gene or triple-gene mutations could partly relieve glucose repression, thereby promoting maltose metabolism and the leavening ability of baker’s yeast in lean dough.

**Conclusions:**

The mutants of industrial baker’s yeast with enhanced maltose metabolism and leavening ability in lean dough were developed by genetic engineering. These baker’s yeast strains had excellent potential industrial applications.

## Background

Baker’s yeast (*Saccharomyces cerevisiae*) is the key microorganism used in the baking industry. Although a small amount of free sugars exists in lean dough with no added sugar, maltose represents the principal source of fermentable carbon during dough fermentation [[1]–[3]]. A good baker’s yeast should rapidly ferment maltose. However, glucose and fructose are the first sugars to be used during fermentation, and the presence or uptake of glucose has a negative impact on the metabolism of other carbon sources [[4]–[7]]. Given that the genes involved in maltose utilization are repressed by glucose, a reasonable way to improve maltose metabolism and leavening ability of baker’s yeast is by effectively alleviating glucose repression.

Mig1, a Cys_2_His_2_ zinc-finger protein, binds to the promoters of several genes and represses their transcription when glucose is added to the medium [[8]–[10]]. Hu et al. have shown that Mig1p represses the transcription of all three *MAL* genes essential to maltose metabolism by binding the upstream genes [[11]]. In addition, Mig1 inhibits transcription by recruiting the general co-repressor complex Ssn6-Tup1 [[12]]. Ssn6-Tup1 is one of the first co-repressor complexes to be identified. As with other co-repressors, the specificity of repression is determined by sequence-specific DNA binding repressors, which recruit Ssn6-Tup1 to the target gene promoters; these repressors include Mig1 [[13]–[16]]. Therefore, a strong correlation among *MIG1*, *TUP1* and *SSN6* for glucose repression was observed. Previous studies have shown that the maltose metabolism of baker’s yeast could be partly glucose derepressed by *MIG1* single-gene mutant through enhancing the transcription of the *MAL* gene [[17]–[19]]. However, the effect of relieving glucose repression on maltose metabolism of baker’s yeast by silencing *TUP1* and/or *SSN6* remains unclear. Furthermore, maltose metabolism of baker’s yeast through combination mutations of *MIG1*, *TUP1* and *SSN6*, which breaking the regulatory pathway of glucose repression, remains unclear.

In this study, we disrupted the regulatory pathway of glucose repression by deleting *MIG1* and/or *TUP1* and/or *SSN6* to investigate the effects of *MIG1*, *TUP1* and *SSN6* on maltose metabolism and leavening ability of baker’s yeast. The results explicitly suggest that the deletion of the *MIG1* and/or *TUP1* and/or *SSN6* genes lead to different results in the tested conditions. Deletion of *MIG1* and/or *SSN6* is more efficient than *TUP1* deletion and other combination deletions of *MIG1*, *TUP1* and *SSN6* on glucose derepression for maltose metabolism and leavening ability of baker’s yeast in lean dough. This finding lays a foundation for the optimization of industrial baker’s yeast strains.

## Results

### Sugar consumption of single-gene deletion strains in low sugar model liquid dough (LSMLD) medium

**Figure 1:**
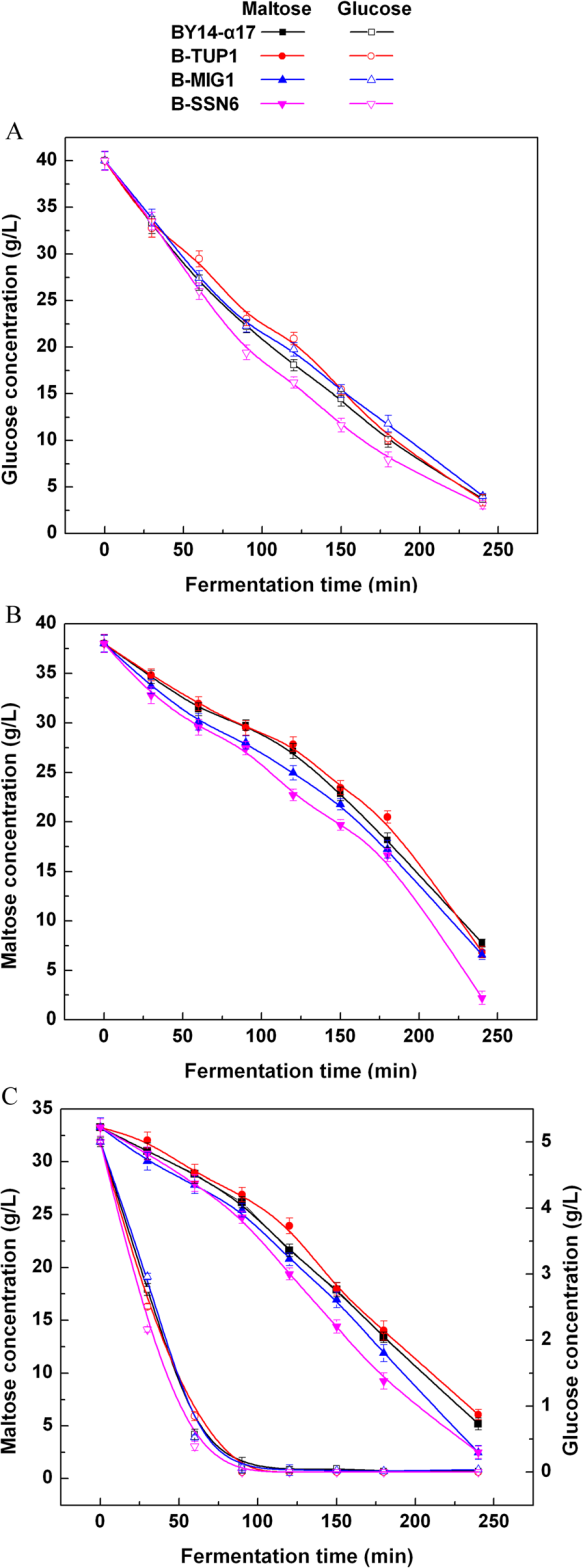
**Concentration of residual sugar in parental strain and single-gene mutants in LSMLD medium.** Fresh yeast cells were inoculated into **(A)** glucose LSMLD medium, **(B)** maltose LSMLD medium and **(C)** glucose-maltose LSMLD medium, and were sampled at suitable intervals. Data are average of three independent experiments and error bars represent ± SD.

The impact of single-gene mutation of *MIG1*, *TUP1* and *SSN6* on sugar consumption was assayed in three LSMLD media. The *TUP1* single-gene-deletion strain B-TUP1 cannot rapidly utilize maltose and is inferior to the parental strain BY14-α17, when glucose was exhausted in the glucose-maltose LSMLD medium (Figure 1C). Compared with the parental strain BY14-α17, the *MIG1* single-gene-deletion strain B-MIG1 did not evidently change in glucose and maltose LSMLD media (Figures 1A to B). However, compared with the parental strain, a 10.8% increase of maltose utilization efficiency (21.3% in the parental strain and 23.6% in the strain B-MIG1, *P* < 0.05) of the strain B-MIG1 was observed in glucose-maltose LSMLD medium, when glucose was exhausted (Figure 1C). Simultaneously, the time span between the point when half of the glucose and that of the maltose had been consumed decreased by 10.2% compared with the parental strain (Table 1). The utilization efficiency of sugar distinctly increased in the *SSN6* single-gene-deletion strain B-SSN6 compared with the parental strain BY14-α17. The maltose utilization efficiency in B-SSN6 was 18.3% and 19.7% higher than that of the parental strain in maltose (79.6% in the parental strain and 94.2% in the strain B-SSN6, *P* < 0.05) and glucose-maltose (21.3% in the parental strain and 25.5% in the strain B-SSN6, *P* < 0.05) LSMLD media, respectively (Figures 1B to C). Furthermore, compared with the parental strain BY14-α17, the time span in B-SSN6 decreased from 2.15 h to 1.76 h (Table 1).

**Table 1:** Time span of the parental strain and the transformants

**Strains**	BY14-α17	B-MIG1	B-TUP1	B-SSN6	B-MIG1-TUP1	B-MIG1-SSN6	B-TUP1-SSN6	B-MIG1-TUP1-SSN6
**Time span (h)**^**a**^	2.15 ± 0.10	1.93 ± 0.11*	2.22 ± 0.12*	1.76 ± 0.13*	2.07 ± 0.09	1.92 ± 0.14*	2.30 ± 0.10*	2.32 ± 0.11**

These results demonstrate that the single-gene deletion of the three genes (*MIG1*, *TUP1* and *SSN6*) resulted in different effects on the alleviation of glucose repression in the maltose utilization of baker’s yeast. Single-gene deletions of *SSN6* and *MIG1* promote the glucose derepression. Particularly, the single-gene deletion of *SSN6* was more effective than the *MIG1* single-gene deletion. However, *TUP1* single-gene deletion was negative to relieve glucose repression to promote the maltose metabolism.

### Sugar consumption of double-gene deletion strains in LSMLD medium

**Figure 2:**
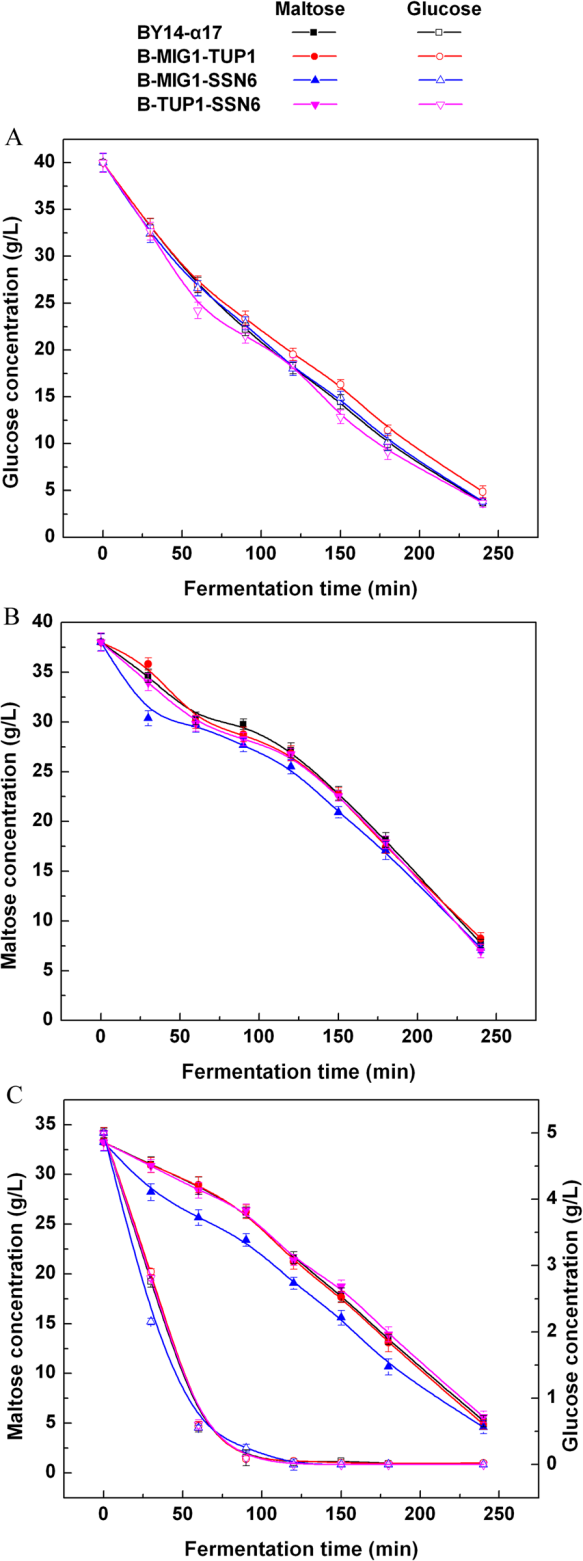
**Concentration of residual sugar in parental strain and double-gene mutants in LSMLD medium.** Fresh yeast cells were inoculated into **(A)** glucose LSMLD medium, **(B)** maltose LSMLD medium and **(C)** glucose-maltose LSMLD medium, and sampled at suitable intervals. Data are average of three independent experiments and error bars represent ± SD.

The maltose metabolism was tested for the double-gene mutants of *MIG1*, *TUP1* and *SSN6* in the LSMLD medium. Although glucose and maltose decreased with BY14-α17 in the strains B-MIG1-TUP1 and B-TUP1-SSN6 in the three LSMLD media (Figure 2), the time span of B-TUP1-SSN6 was still 6.98% higher than the parental strain (Table 1). By contrast, a positive effect with decreased time span (10.7%) was obtained in B-MIG1-SSN6 (Table 1). When the yeast cells were inoculated in the maltose and the glucose-maltose LSMLD media, the strain B-MIG1-SSN6 exhibited a substantially more rapid sugar-uptake than the other strains. Compared with the parental strain BY14-α17, maltose utilization efficiency (21.3% in the parental strain and 29.7% in the strain B-MIG1-SSN6, *P* < 0.05) distinctly increased by 39.4% in the strain B-MIG1-SSN6, when glucose was exhausted in the glucose-maltose LSMLD medium (Figure 2C).

These results indicate that the double-gene deletion of the three genes (*MIG1*, *TUP1* and *SSN6*) also generated different effects on the maltose metabolism of baker’s yeast by alleviating glucose repression. The co-gene-deletion of *MIG1* and *SSN6* mitigated glucose repression, which is more efficient than *MIG1*-*TUP1* and *TUP1*-*SSN6* double-gene deletions with no evident function in maltose metabolism.

### Sugar consumption of the triple-gene deletion strain in LSMLD medium

**Figure 3:**
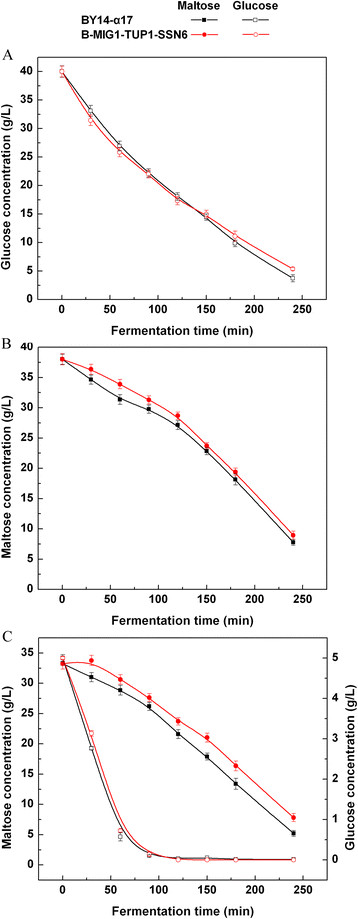
**Concentration of residual sugar in parental strain and triple-gene mutants in LSMLD medium.** Fresh yeast cells were inoculated into **(A)** glucose LSMLD medium, **(B)** maltose LSMLD medium and **(C)** glucose-maltose LSMLD medium, and sampled at suitable intervals. Data are average of three independent experiments and error bars represent ± SD.

The maltose metabolism was further investigated with B-MIG1-TUP1-SSN6, which performs the triple-gene-deletion of *MIG1*, *TUP1* and *SSN6*. Surprisingly, in B-MIG1-TUP1-SSN6, the maltose uptake was considerably delayed compared with the parental strain BY14-α17 until the termination of the process in the maltose LSMLD medium (Figure 3B). Compared with the parental strain, the maltose utilization efficiency (21.3% in the parental strain and 16.9% in the strain B-MIG1-TUP1-SSN6, *P* < 0.05) of the strain B-MIG1-TUP1-SSN6 decreased by 20.7% in the glucose-maltose LSMLD medium (Figure 3C). The consumption of maltose was slower than BY14-α17 throughout the process. Moreover, the time span was evidently increased (from 2.15 h to 2.32 h) (Table 1).

These results suggest that the *MIG1*, *TUP1* and *SSN6* triple-gene deletions were unavailable to relieve glucose repression and enhance maltose metabolism of baker’s yeast, though *MIG1*, *TUP1* and *SSN6* could function as a complex that affects the glucose-repressible genes [[12]].

### Growth and fermentation properties

**Figure 4:**
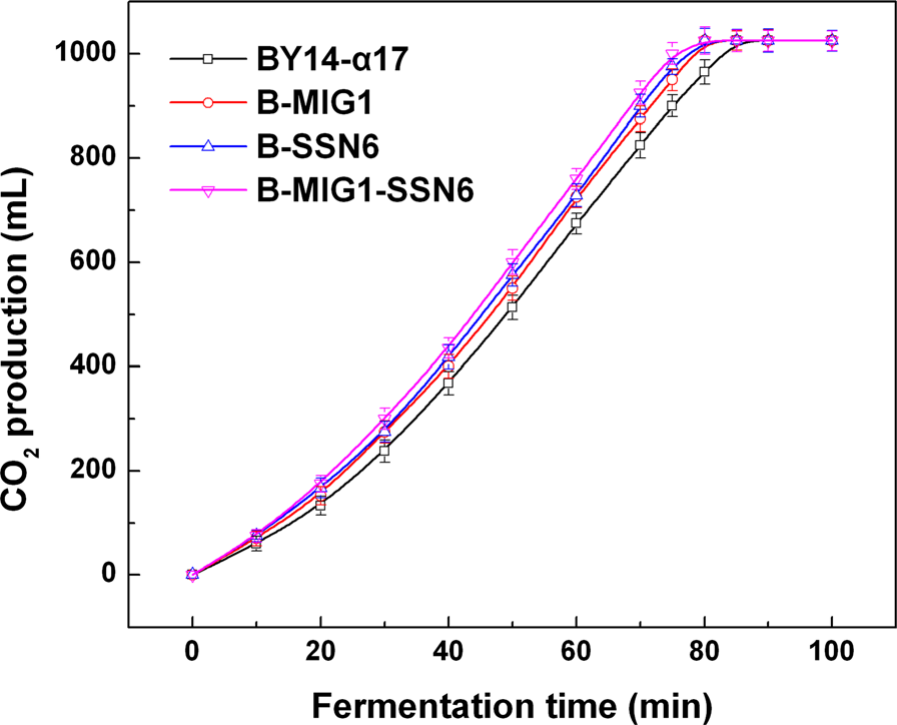
**CO**_**2**_**production by parental strain and positive mutants in lean dough.** We mixed 280 g of flour, 150 mL of water, 4 g of salt, and 8 g of fresh yeast into a fermentograph until steady gas formation was achieved. Data are average of three independent experiments and error bars represent ± SD.

Considering the diversity of sugars consumption of the eight strains obtained, we further investigated the growth characteristics (specific growth rate and biomass yield) under different carbon sources and explored the leavening ability in lean dough. The specific growth rate and biomass yield of the single-gene-deletion and double-gene-deletion strains illustrated in Table 2 comparatively remained stable (small difference with no statistical significance), revealing that the single-gene and double-gene deletions of *MIG1*, *TUP1* and *SSN6* did not influence the growth of the strains. However, the specific growth rate of the triple-gene-deletion B-MIG1-TUP1-SSN6 (0.14 h^−1^) was lower than that of the parental strain BY14-α17 (0.19 h^−1^) in the maltose LSMLD medium. Compared with the parental strain, the biomass yield of B-MIG1-TUP1-SSN6 decreased from 5.9 g/L to 5.1 g/L in the glucose-maltose LSMLD medium (Table 2). The positive mutants B-MIG1, B-SSN6 and B-MIG1-SSN6 performed well for the leavening ability. Compared with the parental strain BY14-α17, the amount of evolved CO_2_ by B-MIG1, B-SSN6 and B-MIG1-SSN6 within 70 min increased from 825 mL to 875 mL (*P* < 0.05), 900 mL (*P* < 0.01) and 925 mL (*P* < 0.01), respectively (Figure 4), while the other strains (B-TUP1, B-MIG1-TUP1, B-TUP1-SSN6 and B-MIG1-TUP1-SSN6) showed lower CO_2_ production (data not shown). Moreover, the fermentation time in B-MIG1, B-SSN6 and B-MIG1-SSN6 were evidently shortened, compared with the parental strain.

**Table 2:** Growth properties of the parental strain and the transformants

**Strains**	**Specific growth rate (h**^**−1**^**)**			**Biomass yield (g/L)**		
	**Glucose**	**Maltose**	**Glucose-maltose**	**Glucose**	**Maltose**	**Glucose-maltose**
BY14-α17	0.19 ± 0.01	0.19 ± 0.00	0.16 ± 0.02	6.2 ± 0.20	6.1 ± 0.21	5.9 ± 0.18
B-MIG1	0.16 ± 0.03	0.18 ± 0.02	0.17 ± 0.02	5.9 ± 0.23	6.0 ± 0.19	6.0 ± 0.20
B-TUP1	0.17 ± 0.02	0.16 ± 0.03	0.16 ± 0.01	5.9 ± 0.18	5.8 ± 0.23	5.9 ± 0.23
B-SSN6	0.21 ± 0.02	0.17 ± 0.01	0.18 ± 0.01	6.3 ± 0.21	6.1 ± 0.23	6.1 ± 0.21
B-MIG1-TUP1	0.16 ± 0.01	0.15 ± 0.04	0.18 ± 0.03	6.0 ± 0.22	5.9 ± 0.21	6.0 ± 0.21
B-MIG1-SSN6	0.17 ± 0.02	0.18 ± 0.01	0.18 ± 0.02	5.8 ± 0.21	6.0 ± 0.20	6.1 ± 0.20
B-TUP1-SSN6	0.20 ± 0.03	0.20 ± 0.02	0.15 ± 0.00	6.3 ± 0.19	6.2 ± 0.22	5.8 ± 0.18
B-MIG1-TUP1-SSN6	0.16 ± 0.02	0.14 ± 0.05*	0.16 ± 0.01	6.0 ± 0.21	5.8 ± 0.20	5.1 ± 0.24*

Values shown represent averages of at least three independent experiments (data are means ± SD). Significant difference of B-MIG1-TUP1-SSN6 from the parental strain was confirmed by Student’s *t*-test (*P < 0.05, n = 3).

These fermentation findings directly correspond with sugar consumption in the LSMLD medium suggesting that the deletion of *MIG1* and/or *TUP1* and/or *SSN6* led to different effects on the leavening ability of baker’s yeast in lean dough. Particularly, *MIG1* and/or *SSN6* deletions could improve the fermentation with stable physiological characteristics.

## Discussion

Glucose has dramatic down-regulating effects on the metabolism of other sugars and on the leavening properties of baker’s yeast strains. Glucose does not repress all glucose-repressible genes in a similar manner [[20]]. Numerous reports have shown that the Mig1 repressor and Ssn6-Tup1 co-repressor are central components of the glucose repression machinery involved in the regulation of *MAL* expression [[12],[21],[22]]. Moreover, *MIG1* deletion does not alleviate glucose repression of maltose utilization [[11],[23]]. Probably, the genetic backgrounds of the *S. cerevisiae* strains used led to the differences in maltose consumptions of the cells. *TUP1* and *SSN6* mutants produce various phenotypes, including constitutive derepression of numerous glucose-repressible genes, calcium-dependent flocculation, mating-type defects in MATα cells and non-sporulation of homozygous diploids [[24],[25]]. However, the different combinations of mutated *MIG1*, *TUP1* and *SSN6* have never been conceived to improve the maltose metabolism. In this study, the deletion mutations of *MIG1* and/or *TUP1* and/or *SSN6* were established for the industrial baker’s yeast cells, explicitly demonstrating that the deletion of *MIG1* and/or *TUP1* and/or *SSN6* presented different effects on the maltose metabolism and leavening ability of baker’s yeast.

Compared with the parental strain, glucose repression of maltose metabolism was partly alleviated by *MIG1* single-gene deletion in B-MIG1 strain (Figures 1B to C), supporting the point that the disruption of *MIG1* causes partial alleviation of glucose repression by the secreted metabolites [[11],[22]]. Surprisingly, an apparent difference was observed between the two members of the co-repressor Ssn6-Tup1, *SSN6* and *TUP1*. The trend for sugar consumption suggests that the maltose metabolism in *TUP1* single-gene-deletion strain B-TUP1 was longer than the parental strain BY14-α17, pointing to a negative alleviation of glucose repression. In contrast, glucose repression was partially relieved in *SSN6* single-gene-deletion strain B-SSN6, increasing maltose metabolism (Figures 1B to C). The different functional domains of Tup1 and Ssn6 involved in glucose control are probably the major causes of the different effects of the two gene deletions on glucose repression. The functional domains of Ssn6 primarily consist of 10 tandem copies of a TPR motif and are specifically necessary for the repression of glucose-regulated genes [[26]]. Different domains of Tup1 can cause the repression of different target genes. For example, some WD motifs or N-terminus domains of Tup1 are not essential for repression of genes regulated by glucose [[26]–[29]]. However, certain regions of Tup1 could be necessary for the high-level expression of glucose-repressed genes, such as *GAL* genes for galactose fermentation [[30]]. Thus, we propose that the regions of Tup1 crucial to the expression of *MAL* genes are disrupted through complete gene deletion. Therefore, *MIG1* single-gene deletion and *SSN6* single-gene deletion were considered effective in improving maltose metabolism in the industrial baker’s yeast.

The combined effects of *MIG1*, *SSN6* and *TUP1* on the maltose metabolism in industrial baker’s yeast were further investigated. Combined mutations of *TUP1* with *MIG1* or *SSN6* compensated for the slow maltose metabolism of the strain B-TUP1, while the rates of maltose consumption in the mutants B-MIG1-TUP1 and B-TUP1-SSN6 were only close to that of the parental strain BY14-α17 (Figure 2). *TUP1* single-gene deletion is possible in its negative effect limited to the alleviation of glucose repression. Hence, *MIG1* or *SSN6* deletion with *TUP1* cannot enhance the maltose metabolism. The double-gene mutant strain B-MIG1-SSN6 was less glucose repressed compared with the parental strain (Figure 2C). This finding corresponds with the studies, which showed that the interactions with DNA-binding repressors are mainly mediated through the different surfaces of Ssn6, and that Ssn6 specifically interacts with Mig1 [[31],[32]]. The insufficient alleviation of glucose repression in B-MIG1-SSN6 could result from the co-action of Mig1 and Ssn6. Although Mig1 is unessential for tethering Ssn6 to the *MAL* upstream, it is important for Ssn6-mediated repression in response to glucose. Surprisingly, the triple-gene mutant B-MIG1-TUP1-SSN6 was more glucose repressed than the parental strain (Figure 3). Considering that the interactions of Ssn6-Tup1 complex contain diverse mechanisms, other mechanisms affecting the *MAL* genes expression are also possibly involved in the regulation of the repression by the Mig1-Tup1-Ssn6 complex [[13],[33]]. In addition, the inferior sugar uptake in B-MIG1-TUP1-SSN6 could be caused by the feeble physiological characteristic compared with the parental strain (Table 2).

Single-gene and double-gene deletions did not present any evident changes in the specific growth rate and biomass yield in the three LSMLD media (Table 2). In other words, the growth properties of single-gene and double-gene deletions of *MIG1* and/or *TUP1* and/or *SSN6* with no distinctive difference (no statistical significance) were insufficient to affect the maltose metabolism. Therefore, the differences in maltose metabolism (maltose utilization and CO_2_ production) do not have strong correlations with the indistinct differences in the physiological effects of the baker’s yeast in this study.

The effective alleviation of glucose repression or the rapid transition from glucose to maltose metabolism is essential to improve the leavening ability of baker’s yeast in lean dough. The single-gene *MIG1*/*SSN6* and co-gene-deletions of *MIG1* and *SSN6* decreased the span time in the glucose-maltose LSMLD medium with stable growth properties (Tables 1 and 2). Therefore, these deletions could cause efficient leavening ability (Figure 4). Furthermore, evident increase of the leavening ability level was observed in B-MIG1-SSN6, indicating that Mig1 and Ssn6 collectively act for the inhibition of maltose-utilizing genes. Thus, co-gene deletion of *MIG1* and *SSN6* could significantly enhance leavening ability of baker’s yeast. These advantages are consistent with the requirement for the leavening ability of an industrial baker’s yeast strain. With minimal transformation, *SSN6* or *MIG1* single-gene deletion is necessary to obtain a baker’s yeast strain with rapid maltose metabolism.

## Conclusion

The results of this study show that the glucose repression involved in the maltose metabolism can be modulated at different levels through the different mutations of *MIG1* and/or *TUP1* and/or *SSN6*. The deletion of *TUP1* was negative to alleviate glucose repression to facilitate the maltose metabolism. In contrast, deletions of *MIG1* and/or *SSN6* were efficient to relieve glucose repression, therefore, promoting maltose metabolism and the leavening ability of baker’s yeast in lean dough. Hence, such baker’s yeast has excellent commercial and industrial applications.

## Materials and methods

### Strains and vectors

Table 3 summarizes the genetic properties of all strains and plasmids used in this study.

**Table 3:** Characteristics of strains and plasmids used in the present study

**Strains or plasmids**	**Relevant characteristic**	**Reference or source**
**Strains**		
***E. coli*** **DH5α**	Φ80 *lacZ*ΔM15 Δ*lacU169 recA1 endA1 hsdR17 supE44 thi-1 gyrA relA1*	Stratagene
**BY14-α17**	*MAT α*, Industrial baker’s yeast	This study
**B-MIG1**	*MAT α*, Δ*MIG1*:: *loxP*	This study
**B-TUP1**	*MAT α*, Δ*TUP1*:: *loxP*	This study
**B-SSN6**	*MAT α*, Δ*SSN6*:: *loxP*	This study
**B-MIG1-TUP1**	*MAT α*, Δ*MIG1*:: *loxP*, Δ*TUP1*:: *loxP*	This study
**B-MIG1-SSN6**	*MAT α*, Δ*MIG1*:: *loxP*, Δ*SSN6*:: *loxP*	This study
**B-TUP1-SSN6**	*MAT α*, Δ*TUP1*:: *loxP*, Δ*SSN6*:: *loxP*	This study
**B-MIG1-TUP1-SSN6**	*MAT α*, Δ*MIG1*:: *loxP*, Δ*TUP1*:: *loxP*, Δ*SSN6*:: *loxP*	This study
**Plasmids**		
**pUG6**	*E. coli/S. cerevisiae* shuttle vector, containing *Amp*^*+*^ and *loxP-kanMX-loxP* disruption cassette	[[34]]
**pUC19**	Ap^r^, cloning vector	Invitrogen
**pSH-Zeocin**	Zeo^r^, Cre expression vector	[[35]]
**pKAB**	Ap^r^, Kan^r^, A_m_-*KanMX*-B_m_	[[10]]
**pUC-KA**_**t**_**B**_**t**_	Ap^r^, Kan^r^, A_t_-*KanMX*-B_t_	This study
**pUC-KA**_**s**_**B**_**s**_	Ap^r^, Kan^r^, A_s_-*KanMX*-B_s_	This study

### Growth, cultivation and fermentation conditions

Recombinant DNA was amplified in *Escherichia coli* DH5α, which was grown at 37°C in Luria–Bertani medium (10 g/L tryptone, 5 g/L yeast extract, and 10 g/L NaCl) supplemented with 100 μg/mL ampicillin. The plasmid was obtained using a Plasmid Mini Kit II (D6945, Omega, USA).

The yeast strains were maintained in yeast extract peptone dextrose (YEPD) medium (10 g/L yeast extract, 20 g/L peptone, and 20 g/L glucose) at 30°C. Over the next enrichment of the molasses medium, cells were harvested through centrifugation (4°C, 1500 ×g, 5 min) and were washed twice with sterile water at 4°C in the succeeding fermentation experiments. To investigate the degree of repression between the three repression factors under different concentrations of extracellular maltose, we used the low sugar model liquid dough fermentation medium [LSMLD fermentation medium, 2.5 g/L (NH_4_)_2_SO_4_, 5 g/L urea, 16 g/L KH_2_PO_6_, 5 g/L Na_2_HPO_4_, 0.6 g/L MgSO_4_, 0.0225 g/L nicotinic acid, 0.005 g/L Ca-pantothenate, 0.0025 g/L thiamine, 0.00125 g/L pyridoxine, 0.001 g/L riboflavin, and 0.0005 g/L folic acid], containing one of the three specified carbon sources (40 g/L glucose, 38 g/L maltose, and 33.25 g/L maltose mixed with 5 g/L glucose).

To select Zeocin-resistant yeast strains, 500 mg/L Zeocin (Promega, Madison, United States) was added to the YEPD plates for the yeast culture. Then, the YEPG medium (10 g/L yeast extract, 20 g/L peptone, and 20 g/L galactose) was used for *Cre* expression in the yeast transformants.

### Construction of plasmid and yeast transformants

Genomic yeast DNA was prepared from the industrial baker’s yeast strain BY14-α17 using a yeast DNA kit (D3370-01, Omega, USA). The PCR primers used in this work are listed in Table 4.

**Table 4:** Primers used in the present study (restriction sites are italics)

**Primers**	**Sequence (5’ → 3’)**
**For plasmid construction**	
TA-U	CCG*GAATTC*AAATGAAATAATACGGGAAGAGCG
TA-D	CGG*GGTACC*CGGTAGCGATAATGTAAGAGGGTT
TB-U	ACGC*GTCGAC*GAACAGAACACAAAAGGAACAC
TB-D	ACAT*GCATGC*GAACCGCAATATTCAGAAACAC
SA-U	CCG*GAATTC*CTTATAACGTGGGCCATGTCAT
SA-D	CGC*GGATCC*CTAGTGACGTTGTCGTATTTGG
SB-U	CGC*GGATCC*TCAACGAGAAATGTTGTGTAGC
SB-D	CCC*AAGCTT*ACATATGCTCATCGGGAAAACC
Kan-U	CGC*GGATCC*CAGCTGAAGCTTCGTACGC
Kan-D	CGC*GGATCC*GCATAGGCCACTAGTGGATCTG
**For PCR verification**	
YT-U	TCTTGTCTGTCTGCTTCTTCACTGT
YT-D	AAAGAGTGTGAAGTGACGGCTATG
YS-U	CACACTCCGTTCTTAGTGGTTGTT
YS-D	ATCCACCGTAGAACCCAAAGCATT
K-U	CTTGCTAGGATACAGTTCTCACATCA
K-D	CGCATCAACCAAACCGTTATTCATTC
Z-U	CCCACACACCATAGCTTCA
Z-D	AGCTTGCAAATTAAAGCCTT

An upstream homologous fragment of the *TUP1* gene was amplified by PCR using BY14-α17 genomic DNA as template with TA-U and TA-D primers. A downstream homologous fragment was similarly amplified using TB-U and TB-D primers. Then, the PCR products were digested using the appropriate endonucleases and were cloned to the pUC19 cloning vector at *EcoR* I and *Kpn* I sites, and *Sal* I and *Sph* I sites, respectively, to construct plasmid pUC-A_t_B_t_. The *KanMX* cassette, which was amplified by PCR using pUG6 as the template with the primers Kan-U and Kan-D, was cloned to construct pUC-A_t_B_t_ after being digested with the appropriate endonucleases to produce the final plasmid, which was designated as pUC-KA_t_B_t_. Based on the aforementioned strategy, the pUC-KA_s_B_s_ plasmid was constructed by inserting the upstream homologous fragment *SSN6*A, *KanMX* cassette, and downstream homologous fragment *SSN6*B into the pUC19 cloning vector.

Baker’s yeast transformation was achieved through lithium acetate/PEG method [[36]]. The deletion cassette of *TUP1*A-*loxP*-*KanMX*-*loxP*-*TUP1*B was amplified and transformed into the industrial baker’s yeast BY14-α17. The fragment was integrated into the chromosome at the *TUP1* locus of BY14-α17 by homologous recombination to construct the *TUP1* deletion strain. The selection of *TUP1* deletion strain was performed using the YEPD medium supplemented with 800 mg/L G418. After selection, recombinant strains were verified with the primers listed in Table 4. Cre recombinase was expressed and *KanMX* was excised after introducing the plasmid pSH-Zeocin into the *TUP1* deletion strain, thus resulting in B-TUP1. The same procedure was utilized to construct B-MIG1 and B-SSN6. Based on the aforementioned strategy, the double-gene mutation strains B-MIG1-TUP1, B-MIG1-SSN6 and B-TUP1-SSN6 were constructed by transforming the second deletion cassette into the single-gene mutation strains. The triple-gene mutation strain B-MIG1-TUP1-SSN6 was constructed by transforming *SSN6*A-*loxP*-*KanMX*-*loxP*-*SSN6*B into B-MIG1-TUP1. Finally, all of the transformants were verified through PCR with the primers listed in Table 4.

### Determination of specific growth rate and biomass yield

After incubating for 24 h, the mixtures of cell culture and medium were mixed in a specific pore plate in appropriate proportions, and the growth curve was detected using bioscreen automated growth curves (Type Bioscreen C, Finland). The specific growth rate was determined with the ratio of the growth velocity to cell concentration.

Nitrocellulose filters with a pore size of 0.45 mm (Gelman Sciences, Ann Arbor, MI, USA) were pre-dried in a microwave oven at 150 W for 10 min and were subsequently weighed. Harvested cells were obtained from 10 mL of cell culture, washed twice with isometric distilled water, and dried at 105°C for 24 h. The biomass yield was determined from the slopes of the plots of biomass dry weight versus the amount of consumed sugar during exponential growth. Experiments were conducted at least thrice.

### Determination of leavening ability

The leavening ability of yeast cells was assayed by measuring the CO_2_ production in lean dough. Lean dough consisted of 280 g of flour, 150 mL of water, 4 g of salt, and 8 g of fresh yeast. The dough was evenly and quickly mixed for 5 min at 30 ± 0.2°C, and placed inside the box of a fermentograph (Type JM451, Sweden). CO_2_ production was recorded at 30°C. Experiments were conducted at least thrice.

### Analysis of sugar consumption

For extracellular sugar measurements, cultures were sampled at 30°C at suitable intervals for 4 h. The maltose content was measured through 3,5-dinitrosalicylic acid method (DNS). HPLC with a refractive index detector and an Aminex® HPX-87H column (Bio-Rad, Hercules, CA, USA) was utilized at 65°C with 5 mM H_2_SO_4_ as the mobile phase at a flow rate of 0.6 mL/min [[37]] to analyze the mixed sugars filtered through 0.45 μm pore size cellulose acetate filters (Mil-lipore Corp, Danvers, MA, USA). The maltose utilization efficiency in maltose LSMLD medium was determined by the ratio of the consumed maltose in 240 min and the total maltose. The maltose utilization efficiency in glucose-maltose LSMLD medium was determined by the ratio of the consumed maltose, when glucose was exhausted, and the total maltose. Based on the consumption curves of glucose and maltose in the glucose-maltose LSMLD medium, the time span between the point when half of the glucose and that of the maltose had been consumed was determined. Experiments were conducted at least thrice.

### Statistical analysis

Data were expressed as mean ± SD, and were accompanied by the number of experiments independently performed. The differences of the transformants compared with the parental strain were confirmed by Student’s *t*-test. Differences at *P <* 0.05 were considered significant differences in statistics.

## Competing interests

The authors declare that they have no competing interests.

## Authors’ contributions

XL carried out the experiments and drafted the manuscript. XWB and HYS participated in the plasmid and strain construction. CYZ and DGX conceived the study and reviewed the final manuscript. All authors read and approved the final manuscript.
